# Secondary Analysis of Interstitial Cystitis/Bladder Pain Syndrome Patients Enrolled in a Recurrent Urinary Tract Infection Prevention Study Provides a Novel Paradigm for Etio-Pathogenesis and Practical Management of This Infection Phenotype

**DOI:** 10.3390/pathogens13050396

**Published:** 2024-05-09

**Authors:** J. Curtis Nickel, Tiziana Cotechini, R. Christopher Doiron

**Affiliations:** 1 Department of Urology, Queen’s University, Kingston, ON K7L2V7, Canada; chris.doiron@queensu.ca; 2 Department of Biomedical and Molecular Sciences, Queen’s University, Kingston, ON K7L3N6, Canada

**Keywords:** urinary tract infection, interstitial cystitis, bladder pain syndrome, recurrent urinary tract infection, vaccine, women’s health

## Abstract

Introduction: A subset of interstitial cystitis/bladder pain syndrome (IC/BPS) patients experience recurrent urinary tract infection (rUTI) associated with symptom flares. Recurrent UTI subjects with associated IC/BPS were enrolled in the first North American early clinical experience trial evaluating a new sublingual UTI preventative vaccine, MV140. It has been shown that women with rUTI develop an imbalance in the T helper 1 and 2 (Th2 over-expression) in the bladder mucosa. Our hypothesis-generating secondary analysis will suggest that this infection subcategory of IC/BPS patients develop a similar imbalance of Th1-Th2 response type to bacteria present in their urinary microbiome, leading to a bladder hypersensitivity that responds to mucosal immune modulation. Methods: Female participants with ≥3 documented UTI/year underwent a 3-month vaccination treatment period with a 9-month efficacy period after completion of vaccine treatment (total 12 months). There were no exclusion criteria for subjects in relation to baseline urinary symptoms and/or discomfort/pain. Primary outcome was no UTI following vaccination. Secondary outcomes included change in UTI incidence, overall patient-reported subjective global assessment (SGA responder defined as moderately or markedly improved on 7-point scale), and safety. Results: Sixteen subjects with IC/BPS-related symptoms and rUTI (mean age 47; range 23–74 years; mean number of UTI episodes in previous year 6.1 +/− 4.2) were eligible to be included in the Health Canada-approved MV140 vaccine study for prevention of rUTI. All subjects completed the 3-month vaccination period. One subject was lost to follow-up after their 6-month visit. Six subjects were UTI-free, while all 16 subjects had a reduction in UTI episodes compared to the year pre-vaccination. The total post-vaccination reduction in UTI episodes compared to pre-vaccination was 80% (0.1 UTI/subject/month from 0.5 UTI/subject/month, respectively). At 12 months, 13 subjects (81%) were SGA responders (moderately or markedly improved), and the responders reported a reduction in IC/BPS symptoms, with 8 subjects reporting significant or almost complete resolution of their specific long-term bladder discomfort/pain and bothersome urinary frequency or urgency. Four subjects reported mild and self-limited adverse events during vaccination period, but none were related to MV140 vaccine. Conclusion: Sublingual MV140 vaccine in IC/BPS patients with rUTI not only achieved UTI-free or reduced UTI incidence status but also, after approximately 9 months post vaccination, resolution of patients’ long-term treatment-refractory IC/BPS symptoms. This suggests some cases of IC/BPS may be etiologically based on Th2-driven hypersensitivity to bacteria within or entering the urinary microbiome that responds to a vaccine whose mechanism of action is to normalize or balance the bladder Th1/Th2 mucosal immune system.

## 1. Introduction

Interstitial cystitis/bladder pain syndrome (IC/BPS) is characterized by bladder pain and storage symptoms (urinary urgency and frequency) [1]. The diagnosis is made clinically by the specific symptom complex and exclusion of confusable diseases that could mimic the clinical syndrome, including concurrent urinary tract infection (UTI) [2]. It is acknowledged that IC/BPS is a prevalent medical condition, particularly in women, but it has been difficult to determine the actual prevalence because definitions and diagnostic criteria (in the absence of a definitive diagnostic test or biomarker) are constantly evolving. It has been estimated that as many as 2.7–6.5% of North American women experience symptoms consistent with a diagnosis of IC/BPS, although fewer than 10% of women who experience these symptoms actually have a diagnosis of IC/BPS [3]. The syndrome does occur in men, with a reported 10:1 female-to-male ratio [4].

To complicate things further, it has been well established that IC/BPS patients exhibit a remarkable prevalence of other chronic overlapping pain conditions (COPCs), such as fibromyalgia, irritable bowel syndrome, chronic back pain, and chronic fatigue syndrome [5,6]. While it has been suggested that the patient population can be described simply as those with bladder/pelvic pain only or those with pain beyond the pelvis (widespread pain) [7,8], it appears clinically that there may be many more clinical phenotypes, each with its own etiological mechanism, symptom complex, and disease trajectory [9]. Each of these “clinical phenotypes” require a unique management strategy to be successful [9,10]. By understanding the potential interrelated etiologies (e.g., autoimmunity, inflammation, urothelial dysfunction, muscular dysfunction, and infection) of the many and various subtypes of IC/BPS with propagation of the symptoms occurring due to complex local neurobiological contributions such as spinal cord crosstalk and central sensitization, we may be able to better treat patients with IC/BPS by individualizing treatment strategies [1,9].

Although similar to chronic UTI symptoms, patients with IC/BPS do not typically complain of dysuria (a hallmark of UTI), and the urine culture, by definition, is negative when employing standard traditional plate culture laboratory testing [11]. However, patients with IC/BPS, particularly women, suffer from the same or more UTIs than those without the diagnosis. In the case of IC/BPS patients, clearing the bacteriuria with appropriate antibiotic therapy does not usually improve the baseline chronic pain symptoms [12]. Many IC/BPS patients describe “symptom flares” when they have a UTI [13], and treatment with antibiotics may bring the symptoms back to baseline again [12]. There is some evidence that long-term, wide-spectrum antibiotic preventative therapy may reduce the pain and voiding symptoms in some IC/BPS patients [14]. Furthermore, experiencing rUTI (and dysfunctional voiding) as a child appears to predispose women to IC/BPS later in life [15]. Multiple analyses of the urinary microbiome (using extended culture or non-culture technology) of women with and without IC/BPS have failed to show either a putative micro-organism or bacterial pathogen pattern responsible for the symptoms [16]. However, some believe that some cases of chronic UTI-like symptoms and/or infection-related cases of IC/BPS may be related to sparce bacterial colonies “embedded” in the bladder mucosa [17]. So, the question remains: Are UTIs, either recurrent or chronic UTI (or even UTI in the past), responsible for the development of the symptom complex we call IC/BPS in at least some patients with an infection IC/BPS phenotype? And if so, what can be done to either eradicate the bacteria and/or ameliorate the symptoms?

MV140, a sublingual polybacterial whole-cell-based vaccine developed for the prevention of rUTI [18,19], has been shown in a pivotal randomized, placebo-controlled trial to decrease the risk of rUTI and even render up to 58% of treated women completely UTI-free in the 9-month post-vaccination efficacy period [20]. A recent Health Canada-approved real-life clinical practice study enrolled women with rUTI, defined as ≥3 UTI in the previous year or ≥2 in the previous 6 months, and treated them with 3 months of MV140 [21]. There were no study criteria to exclude rUTI subjects who had concomitant IC/BPS, as long as they had definitive UTIs (culture-proven) and responded (at least back to baseline pain symptoms) to antibiotics in the previous year. We hypothesize that the response of this cohort of infection phenotype IC/BPS female patients in terms of UTI risk as well as chronic IC/BPS symptoms, along with the mechanism of action of this sublingual immunomodulator, suggests a mucosal immunological etiology as well as a potential therapeutic target.

## 2. Materials and Methods

To be eligible for inclusion in the primary study [21], participants had to be female, <80 years of age, and with ≥3 UTI/year (or ≥2 UTI in the previous 6 months). At least one of the UTIs had to be confirmed with a positive culture with an accepted uropathogen, and all reported UTIs had to have responded to antibiotic therapy based on Canadian Urological Association guideline for the management of rUTI [22]. Since this was the first clinical experience with MV140 vaccine, the study was approved by Health Canada (Biologics and Genetic Therapies Directorate) and conducted in Kingston, ON, Canada (ClinicalTrials.gov: NCT04096820), with approval by the Queen’s University institutional review board (#6026618). The MV140 was supplied in kind for this investigator-initiated study (the official sponsor of the study was the principal investigator, J.C.N.) by Inmunotek S.L. (Spain). The details of the study have been reported elsewhere [21].

While the exclusion criteria included complicated UTIs, comorbidities associated with the genitourinary tract, and/or immunological diseases, there was no exclusion criteria for subjects in relation to baseline urinary symptoms and/or discomfort/pain. Therefore, female subjects with a diagnosis and/or clinical symptoms of IC/BPS and rUTI were enrolled in the study if they met the inclusion criteria.

Eligible subjects (after informed consent obtained and screening completed) were taught to administer MV140 with two sprays daily of 100 µL sublingually for 3 months. MV140 consists of a suspension of whole-cell heat-inactivated bacteria (300 formazin turbidity units; selected strains of four bacterial species—*Escherichia coli* V121, *Klebsiella pneumoniae* V113, *Enterococcus faecalis* V125, and *Proteus vulgaris* V127—at equal percentages) in glycerol, sodium chloride, artificial pineapple flavoring, and water.

Following 3 months of active treatment, subjects were followed-up every 3 months for an additional 9 months. This included the 3-month vaccination period and 9-month efficacy period for a total study duration of 12 months from first dose. Midstream urine cultures were obtained at screening and 12-month visit. Adverse events were reviewed with the subjects at each scheduled visit. A global response assessment (GRA) questionnaire was administered at the end of the efficacy period (12 months).

The current post hoc analysis of the primary study includes those subjects with rUTI identified with the diagnosis of IC/BPS refractory to standard therapy. Similar to the primary real-life clinical practice study, the primary outcome was no UTI following vaccination, where a UTI was clinically defined as acute cystitis-like symptoms requiring a physician prescription of antibiotics for resolution of the acute event. Patients were instructed to obtain a midstream urine culture prior to taking the antibiotic if possible. All UTI reports were adjudicated as a “true clinical” UTI by the investigators. Secondary outcome in this cohort of IC/BPS and rUTI subjects included change in UTI incidence (from pre-vaccination records) and overall patient-reported subjective global response assessment, with responders defined as moderately or markedly improved on a 7-point GRA scale (markedly worse, moderately worse, slightly worse, no change, slightly improved, moderately improved, or markedly improved). Because this is a post hoc analysis, the protocol for the primary study did not include specific IC/BPS questionnaires. Adverse events (AEs) were individually examined to evaluate their severity, intensity, causality, and outcome. Adverse reactions (AR—intervention-related adverse events) were recorded based on location, timing of appearance, intensity, and outcome.

### Data Analysis

This secondary analysis of IC/BPS subjects enrolled in a rUTI non-randomized, unblinded, real-life clinical practice study is completely descriptive and not powered to make any definitive conclusions.

## 3. Results

- Sixty-four subjects (mean age 56 years; range 18–80) completed the 3-month vaccination period and at least one post-vaccination assessment in the primary study. The outcome for this group has been described in the primary publication [21]. Sixteen of these subjects reported unresolved IC/BPS related symptoms and rUTI and were therefore eligible to be included in the MV140 vaccine study for prevention of rUTI. The mean age of this IC/BPS cohort of subjects with rUTI was 47 (range 23–74 years). None of the subjects in this sub-analysis were reported to be the Hunner lesion variant of IC/BPS. The mean number of UTIs (or “UTI episodes) reported in the previous year was 6.1 +/− 4.2. The bacterial organisms reported pre-vaccination in this cohort (two subjects reported two organisms in the previous year) included *E. coli* (9), *Enterococci* sp. (4), *Klebsiella* sp. (3), *Pseudomonas* sp. (1), and *Proteus* sp. (1);
- All 16 IC/BPS-rUTI subjects completed the 3-month vaccination period and were included in both the safety and efficacy population for this secondary analysis. One subject was lost to follow-up after 6-month visit (leaving country); however, a 6-month end-of-study assessment was possible;
- Six subjects (38%) were UTI-free, while all sixteen subjects had a reduction in UTIs compared to the year pre-vaccination. The total post-vaccination reduction in UTIs compared to pre-vaccination was 80% (0.1 UTI/subject/month from 0.5 UTI/subject/month, respectively). At 12 months (end of 9-month efficacy period), 13 subjects (81%) were GRA responders (moderately or markedly improved). Of the three non-responders, two were slightly improved and one was moderately worse. At 12 months, all 13 responders spontaneously reported a reduction in IC/BPS symptoms, with eight subjects reporting significant or almost complete resolution of their specific long-term bladder discomfort/pain and bothersome urinary frequency or urgency (Table 1);
- Eighteen AEs (including one serious AE) were reported in 13 of the 16 subjects (five AEs reported in four subjects during 3-month vaccination period—see Table 2). None of the AEs were suspected to be related to intervention with MV140 vaccine.

## 4. Discussion

Female patients with rUTI and associated chronic bladder pain and storage urinary symptoms between UTI episodes showed a similar beneficial response, in terms of UTI risk, as the entire rUTI population enrolled in the primary study. Subjects with IC/BPS were younger than the entire rUTI population enrolled in the primary study (mean age 47 vs. 56 years); however, the number of UTIs in the previous year was similar (mean number of UTIs 6.1 vs. 6.8). The percentage of subjects who reported no UTI in the efficacy period (post vaccination) was similar in the IC/BPS cohort as that reported for the entire population of rUTI subjects (38% vs. 41%). The reduction in UTIs compared to the number of UTIs reported in the year prior to vaccination was also similar (80% vs. 76%). This small, post hoc analysis of IC/BPS patients with rUTI suggests that rUTI patients will achieve a similar beneficial response to MV140 whether or not they had IC/BPS symptoms in terms of reduction in UTI risk.

The interesting observation was the fact that 13 (out of a total of 16) subjects with IC/BPS evaluated in this secondary analysis who reported a clinically significant global response assessment (GRA responders reporting they were moderately or markedly improved at 12 months) also reported significant improvement of their long-term bladder discomfort/pain and bothersome urinary frequency and urgency. Even more remarkable was that eight of these responders spontaneously reported that they had almost complete resolution of their IC/BPS-like symptoms. This suggests that UTI vaccination might be a safe treatment option for management of IC/BPS patients with rUTI who have been found to be refractory to traditional therapy. 

It has been hypothesized that some IC/BPS or chronic cystitis-like patients may harbor bacteria within the bladder mucosa [17]. Long-term (up to a year or longer has been advocated), wide-spectrum antibiotics to eradicate the possibility of “embedded” pathogens in the bladder mucosa [23] will lead to potential antibiotic adverse effects and change in the normal microbiome in individual patients as well as increase the spectra of community antibiotic resistance. Another option to reduce long-term IC/BPS cystitis symptoms that has recently been popularized is cauterization of observable bladder inflammation (even repeating this procedure as many times as necessary until the patient reports improvement) [24]. Some studies have indicated that this approach has been successful in some patients [25], but it is costly, destructive to the bladder mucosa, and may perhaps have long-term consequences for the bladder wall itself. The administration of a safe sublingual immunomodulator would be a more attractive option.

This analysis suggests that IC/BPS patients with a documented infection phenotype (met the criteria of rUTI) who respond to MV140 in terms of UTI risk reduction may also experience significant amelioration of their IC/BPS symptoms, which for most of the subjects were refractory to standard IC/BPS management strategies. The question that arises from these observations is why a sublingual vaccine developed to reduce rUTI would also ameliorate chronic bladder pain and urinary storage symptoms in women with a diagnosis of both IC/BPS and rUTI. It is very likely that the answer lies in the mechanism of action of this specific sublingual vaccine, MV140.

**Figure 1:**
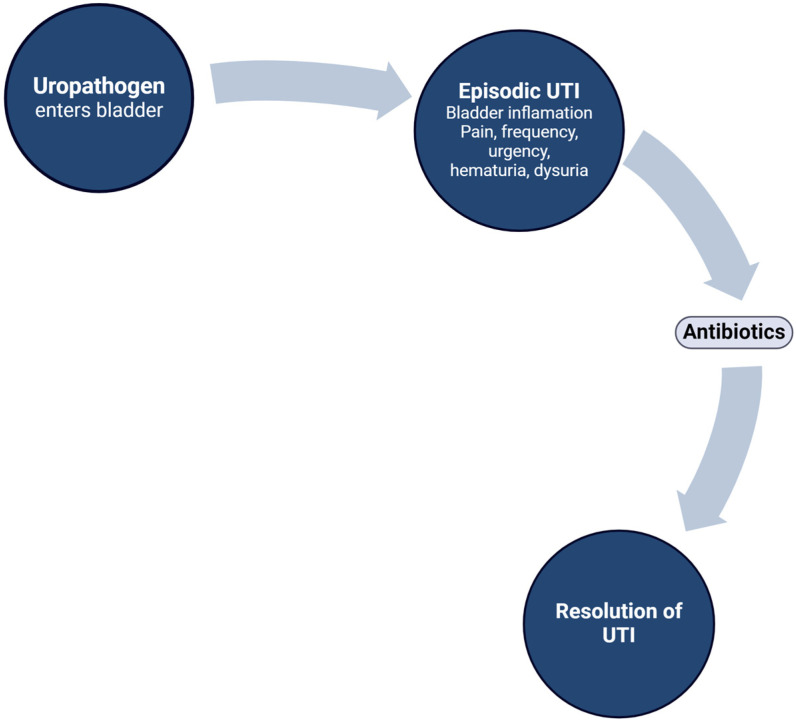
Typical course of an infrequently occurring UTI resulting from uropathogenic bacteria entering the bladder and overcoming the host defenses. The resulting inflammation, pain, and bladder irritability usually resolves with a standard course of an appropriate antibiotic.

For decades, it was accepted that the majority of acute UTI episodes occurring less than several times per year (sometimes only once in a patient’s lifetime) results from a uropathogen entering the urinary tract (typically from the vagina) [26]. The pathogenicity of the microbe overwhelms the host defenses of the bladder, causing acute bladder mucosal inflammation. The resulting pain, storage symptoms, and dysuria usually resolve with appropriate antibiotic therapy [22] (Figure 1).

**Figure 2:**
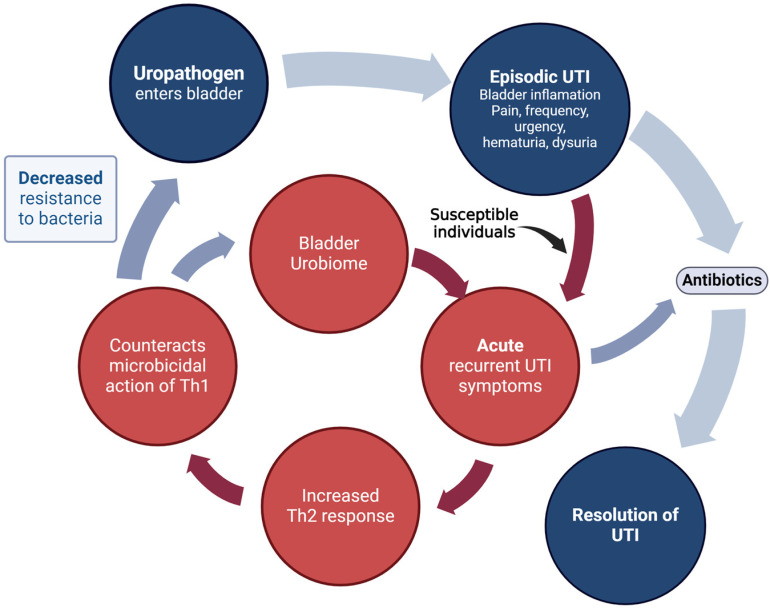
Scenario believed to occur in susceptible females who develop rUTI. In these individuals epithelial damage may result in an increase in T helper 2 (Th2) response in the bladder mucosa, which may counteract Th1-mediated microbicidal action and lead to a cycle of recurrent UTI-like episodes.

However, in some cases, in susceptible women, the epithelial damage results in an increase in T helper 2 (Th2) response [27]. In excess, this Th2 response counteracts Th1-mediated microbicidal action [19] (Figure 2).

**Figure 3:**
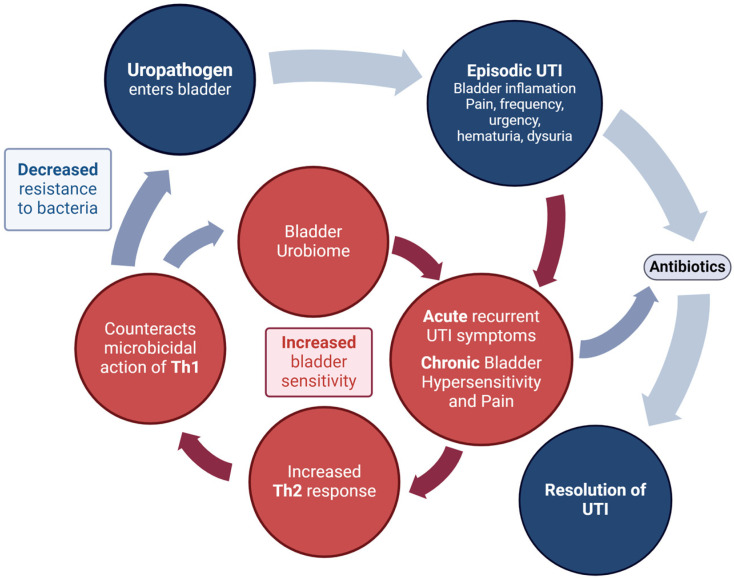
The increased mucosal sensitivity that can occur with the imbalance of Th1:Th2 levels in the bladder mucosa may lead to increased bladder hypersensitivity and pain between rUTI episodes.

These changes can also increase mucosal sensitivity. The end result is increased susceptibility to both exogenous bacteria (from outside the urinary tract) and perhaps even endogenous bacteria (within the patient’s urinary microbiome), creating a cycle of recurrent UTI and, in some patients, bladder hypersensitivity (bladder pain and storage symptoms) (Figure 3).

**Figure 4:**
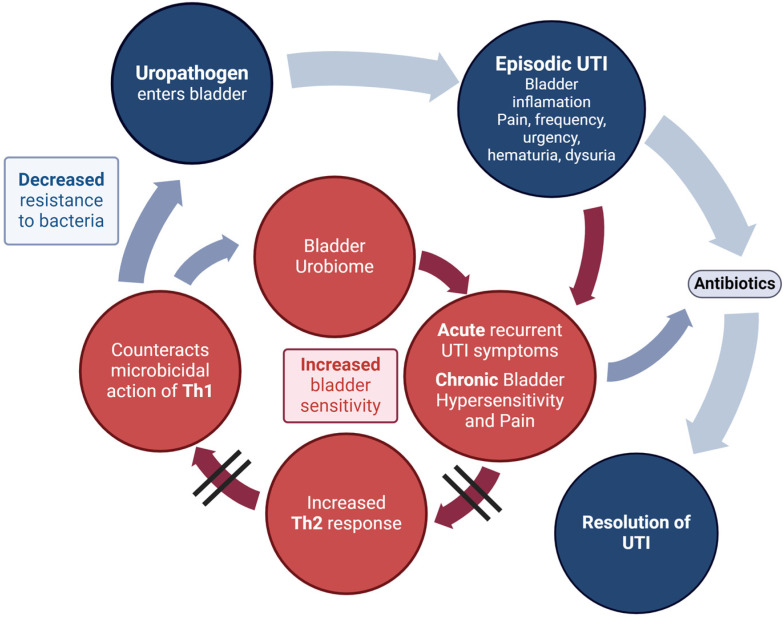
Mechanism by which MV140 decreases risk of rUTI from bacteria outside and inside the bladder microbiome by increasing Th1 and Th17 cells, which both downregulate the abnormal Th2 response and promote or mediate cellular immune responses.

So how does a polyvalent, whole-cell, dead bacterial therapeutic vaccine sprayed under the tongue modulate the immune response to reduce the risk of further UTI in rUTI patients and eventually ameliorate the bladder pain and storage symptoms of patients with an infection phenotype of IC/BPS? The role of bacterial preparations for the prevention of rUTI has been extensively evaluated [28,29,30], and we now understand the potential mechanisms of action of most of these vaccines to induce protective immunity in the bladder [30,31,32]. As described in a previous review of MV140 [19], it appears that the sublingual vaccine induces an immunological change towards normal bladder mucosal immune status in rUTI patients that mediates protection from bacterial pathogens within the bladder [32]. Sublingual MV140 induces antibody production against the bacteria in the vaccine mixture [33] and activates human dendritic cells to generate T helper (Th) 1, Th17, and interleukin-10-producing T-cell responses in secondary lymphoid organs and locally in the bladder [34]. The induction of adaptive immunity memory responses likely underlies the clinical protection observed following treatment discontinuation, although trained immunity (a form of innate immune memory whereby innate immune cells respond to subsequent bacterial triggers with enhanced responsiveness) could also play a role [33,35]. Within the bladder mucosa, MV140 increases Th1 and Th17 cells, which both downregulate the abnormal Th2 response and promote or mediate cellular immune responses, resulting in a decreased risk of rUTI from bacteria outside and inside the bladder microbiome (Figure 4).

**Figure 5:**
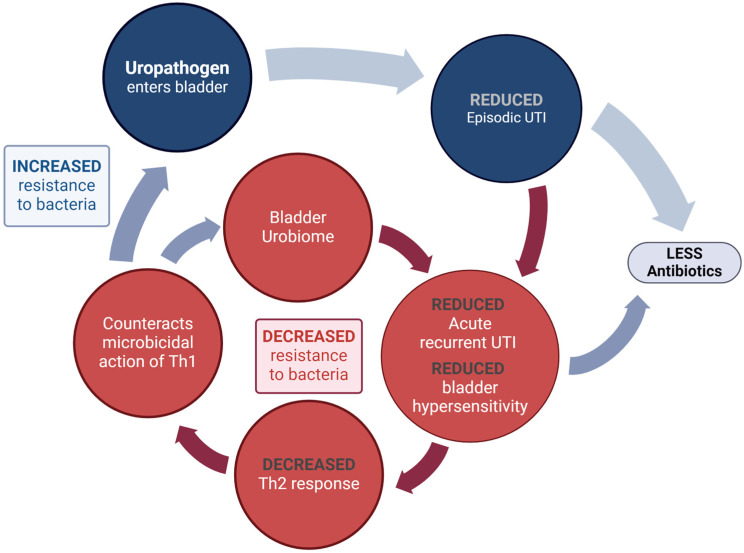
Hypothesis is that restoring the immunological balance within the bladder mucosa in patients with IC/BPS and rUTI will result in eventual amelioration of the bladder mucosal hypersensitivity responsible for the development, propagation, and maintenance of the IC/BPS symptoms.

We further hypothesize that restoring the immunological balance within the bladder mucosa in patients with IC/BPS and rUTI (infection phenotype of IC/BPS) will, over time (as long as the rUTI state has been corrected), result in eventual amelioration of the bladder mucosal hypersensitivity responsible for the development, propagation, and maintenance of the IC/BPS symptoms (Figure 5).

In summary, we hypothesize that this balancing of the bladder mucosal immune system with MV140 not only reduces the risk of further UTI but also, in those patients who have developed bladder hypersensitivity, eventually (up to a year from initiation of vaccine treatment) reverses this symptom complex of bladder pain/discomfort and bladder frequency/urgency.

While there is no doubt that a small cohort of IC/BPS patients with rUTI experienced significant benefit in terms of reduction in both UTI risk and IC/BPS symptoms, there are real limitations to this study and the associated hypotheses. The study is a descriptive post hoc analysis of a small cohort of IC/BPS patients with rUTI enrolled in a primary UTI preventative study. There were no placebo controls in this real-life clinical study, and the protocol did not include specific IC/BPS symptom scoring. Future studies required to examine the hypothesis outlined in this sub-study could include robust immunophenotyping using multiplex spatial imaging and/or single-cell sequencing of IC/BPS mucosal biopsies and prospective randomized, placebo-controlled efficacy studies. Moreover, the use of pre-clinical murine models could be helpful to uncover shared pathogenic mechanisms between rUTI and IC/BPS. The present study, if its observations are confirmed in larger prospective trials, leads to another important question. Although we have shown benefit in IC/BPS patients with documented concurrent condition of rUTI, would all IC/BPS patients with a potential infection phenotype (e.g., UTIs as a child, past history of recurrent UTIs, culture-negative flares that improve with antibiotics, etc.) also experience such a benefit from a vaccine whose mode of action relies on immunological changes within the bladder mucosa? Such an implication may have profound therapeutic value in managing this enigmatic condition we call IC/BPS.

## 5. Summary

We hypothesize that some cases of IC/BPS, i.e., those with an infection phenotype, may be etiologically based on a Th2 cell-type hypersensitivity to bacteria within or entering the urinary microbiome that responds to a vaccine whose mechanism of action is to normalize or balance the bladder Th1/Th2 mucosal immune system. These observations should be confirmed in a properly powered RCT in which female patients with IC/BPS and rUTI are randomized to placebo or MV140 and the impact on IC/BPS symptoms measured with validated IC/BPS related assessment tools.

## 6. Conclusions

IC/BPS patients with rUTI reported UTI-free or reduced UTI incidence status after vaccination with MV140, similar to female patients with rUTI but no IC/BPS diagnosis. Approximately 9 months post vaccination, female patients with IC/BPS and rUTI may experience resolution of their long-term treatment-refractory IC/BPS symptoms.

## Figures and Tables

**Table 1:** Summary tabulation of results at the end of the 9-month efficacy period (12-month visit).

Endpoint	Number (n = 16)	Percentage (%)
UTI-free	6	37.5
UTI reduction	16	100
Total post-vaccination reduction in UTI	0.5 to 0.1 UTI/subject/month	80
Global response assessment responders	13	81
Resolution of IC/BPS symptoms	8	50

**Table 2:** Adverse events reported in 13 subjects. None of the AEs were suspected to be related to study intervention.

Adverse Event	Visit Reported	Outcome
Vaginal yeast infection	3-month	Resolved with probiotics.
Eye infection	3-month	Resolved with local therapy.
Increase in pre-existing non-bladder chronic pain	3-month	Not reported in subsequent visits.
Increase of anxiety symptoms	3-month	Managed with medication.
Light-headedness	3-month	Resolved.
Non-study drug overdose (SAE)	6-month	Hospitalized; resolved.
Mild skin irritation	6-month	Resolved within 1 week with topical steroid.
Vaginal spotting	6- and 9-month	Recurred—managed with hormone therapy.
Increase in pelvic pain and voiding symptoms	6-month	Cystoscopy revealed Hunner lesions; subsequently treated.
Increase in pre-existing depression	6-month	Ongoing through end of study.
Chest pain	9-month	Temporary symptom; no recurrence.
Increase in pre-existing knee pain (SAE)	9- and 12-month	Resolved with knee replacement surgery.
Vaginal yeast infection	12-month	Resolved after antibiotic cessation.
Increase in pre-existing back pain	12-month	Resolved.
Increase in pre-existing depression	12-month	Unknown.
Increase in pre-existing depression	12-month	Resolved with medical treatment.
Increase in GI reflux symptoms	12-month	Unknown.
COVID-19	12-month	Resolved.

## Data Availability

The original report and data are available at reference [21].
